# Bacterial elimination *via* the photodynamic activity of a fullerene/light-harvesting antenna molecule assembled system integrated into liposome membranes†

**DOI:** 10.1039/d0na00132e

**Published:** 2020-08-25

**Authors:** Riku Kawasaki, Daiki Antoku, Reo Ohdake, Kouta Sugikawa, Atsushi Ikeda

**Affiliations:** Department of Applied Chemistry, Graduate School of Engineering, Hiroshima University 1-4-1 Kagamiyama Higashihiroshima 739-8527 Japan

## Abstract

Ease of transmission and exceptionally high mortality rates make pathogen-based infections an ongoing global threat. Herein, a facile bacterial elimination process is described which is based on the photodynamic activity of fullerenes composed of light-harvesting antenna molecules integrated into liposome membranes. This was done to expand the absorption capabilities of fullerene derivatives. Efficient energy transfer from the photoactivated antenna molecules to the fullerenes enhanced antimicrobial activity without any harmful lytic activity against red blood cells even under irradiation.

## Abstract 2

Bacterial elimination was attained by singlet oxygen (^1^O_2_) generated from fullerene derivatives *via* efficient energy transfer from photo-activated light-harvesting antenna molecules.

## Graphical abstract

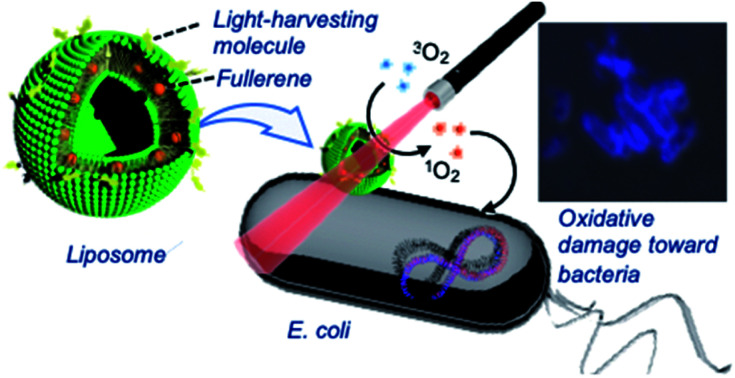

The frequency and severity of serious pathogenic infections globally is of concern because of their rapid transmission rates and high mortality.^1–3^ Since the discovery of penicillin by Dr Fleming in 1928, researchers have developed various types of small molecule antibiotics,^4,5^ peptides,^5,6^ polymers,^7^ and nanomaterials^8^ in an attempt to combat and eliminate this omnipresent threat. The emergence of drug-resistant bacteria due to antibiotic misuse poses a serious risk to global health and safety.^9,10^ Recently, it was revealed that drug resistance can be acquired by *E. coli* after as little as 3 h of exposure.^11^ With this in mind, it is critical that alternative approaches for the elimination of bacteria and other harmful microbes must be developed.^8–10^ Photodynamic therapy (PDT) has been proposed as a candidate for the treatment of cancer,^12–14^ rheumatoid arthritis,^15^ and infections^16^ because of its excellent spatiotemporal selectivity and invasiveness. The therapeutic effects of PDT are achieved through oxidative stress caused by the presence of cytotoxic reactive oxygen species (ROS) such as the singlet oxygen (^1^O_2_) and the superoxide anion (O_2_^−^˙); these are produced by photosensitizers *via* light irradiation.^17^ In comparison with conventional antibiotic courses, PDT has also been proposed as a treatment option for infectious diseases as it requires a relatively short-term treatment course, inflicts severe damage due to oxidative stress, and offers non-specific targeting of the microbial cellular structures and metabolic pathways related to the appropriate receptors.^16^ Of course, the key to successful PDT is highly dependent on the deliverability of the photosensitizers to the right recipient cells.^18^

Many types of photosensitizers have been developed,^18^ including fullerenes,^19^ porphyrins,^20,21^ chlorines,^22,23^ phthalocyanines,^20,23^ and their derivatives. Because of their ability to remain in the excited state for a long time and excellent capacity to generate ROS, fullerenes, and their derivatives are extensively used as possible photosensitizers in PDT.^19^

Despite their fascinating potential for PDT, their bioavailability is oftentimes limited because of poor water solubility and low absorption capacity at longer wavelengths.^24^ Thus, the development of more effective fullerene delivery systems is a necessary step in accessing the full therapeutic potential of these compounds as antimicrobial agents.^25^

To address these issues, we used a liposome as a delivery platform *via* two approaches; in one strategy, the target location could be manipulated through the use of polarized fullerenes (catC_60_), whereas the other approach focused on facilitating more efficient energy transfer from the photo activated antenna molecules to catC_60_ at longer wavelengths. In this study, bacterial elimination was achieved *via* excellent photodynamic activity using a catC_60_/light-harvesting antenna molecule assembly, which was integrated into liposomal membranes (LMIcatC_60_–light-harvesting antenna molecule) (Fig. 1). Natural products, including cyclodextrins,^27^ polysaccharides,^28,29^ and liposomes,^30–32^ were developed based on solubilizing techniques to utilize fullerenes in applications as photosensitizers.^26^ In particular, liposomal membranes are attractive platforms for designing and constructing assembled systems of fullerenes with dialkylated light-harvesting antenna molecules.^32–34^ Previously, the poor absorption capacity of our photosensitizers over the long-wavelength range between 600 and 700 nm was improved and the photoinduced cytotoxicity toward HeLa cells of these types of assembled systems was enhanced. Without the need for complicated synthesis of fullerenes, we used our system, which is based on LMI systems, to maximize and expand the availability of our photosensitizers at will by combining them with commercially available light-harvesting molecules. These results encouraged us to apply our system for bacterial elimination processes.

**Fig. 1:**
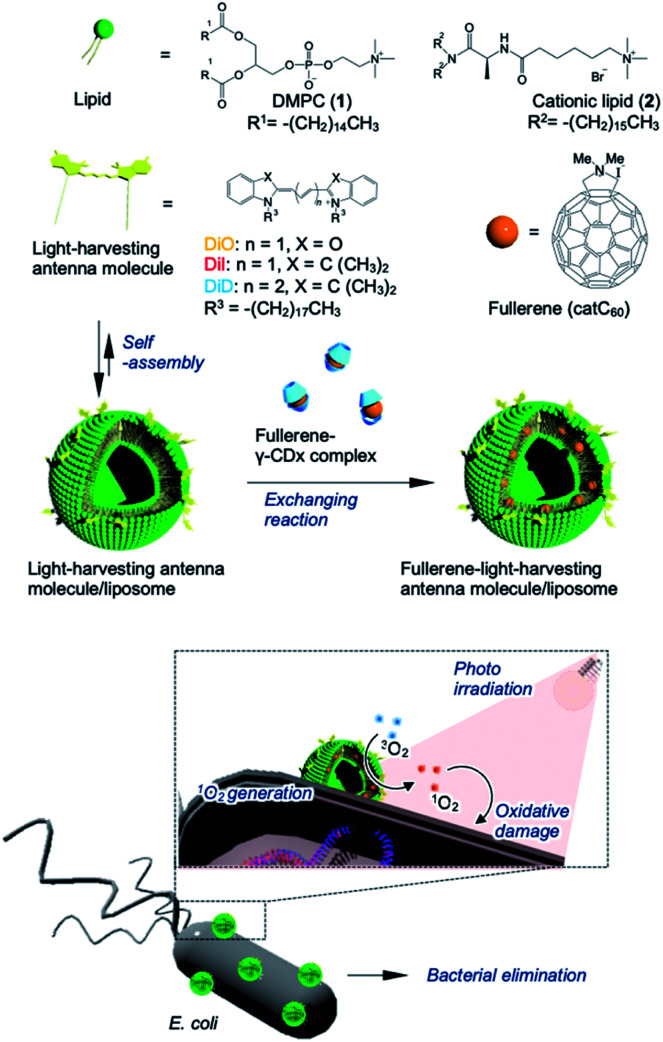
Schematic of bacterial elimination *via* a photodynamic system using LMIcatC_60_–light-harvesting antenna molecules.

The preparation and detailed characterization of the catC_60_/light-harvesting antenna molecule assembled system integrated into the liposomal membranes are reported elsewhere.^33^ Briefly, LMI–light-harvesting antenna molecules were prepared *via* a conventional freeze–thaw method using DMPC and dyes such as 1,1′-dioctadecyl-3,3,3′,3′-tetramethylindocarbocyanine perchlorate (DiI; *λ*_max_, 549 nm) and 1,1′-dioctadecyl-3,3,3′,3′-tetramethylindodicarbocyanine (DiD; *λ*_max_, 644 nm). Afterward, the catC_60_ was introduced into the liposomal membranes *via* an exchange reaction^33^ by mixing the catC_60_/γ-cyclodextrin complex at 80 °C, a temperature which is higher than the phase transition temperature of DMPC (DMPC, 1 mM; DiD or DiI, 0.025 mM; catC_60_, 0.05 mM). The catC_60_/light-harvesting antenna molecule assembled system had a diameter of approximately 80 nm each in cases where DiD and DiI were used (Fig. S1†). When 3,3′-dioctadecyloxacarbocyanine perchlorate (DiO; *λ*_max_, 484 nm) was used, the systems were prepared *via* DMSO injection into LMIcatC_60_ (Scheme S1†) as the dye was unstable at high temperatures (DMPC, 1 mM; DiO, 0.025 mM; catC_60_, 0.05 mM). After the complexation of DiO with LMIcatC_60_, no precipitation was found, and the absorbance from DiO was observed between 400 and 500 nm (Fig. 2A). Furthermore, 94% of the fluorescence signals from DiO were quenched *via* complexation (Fig. 2B and Table 1). This was confirmation that DiO had been successfully introduced into the liposomal membrane *via* the DMSO injection method and that the distance between DiO and catC_60_ facilitated efficient energy transfer. The ζ potential of the liposome decreased slightly as a result of the complexation of DiO from +36 to +30 mV, whereas the hydrodynamic diameter of the liposomes remained relatively unchanged at 80 nm.

**Fig. 2:**
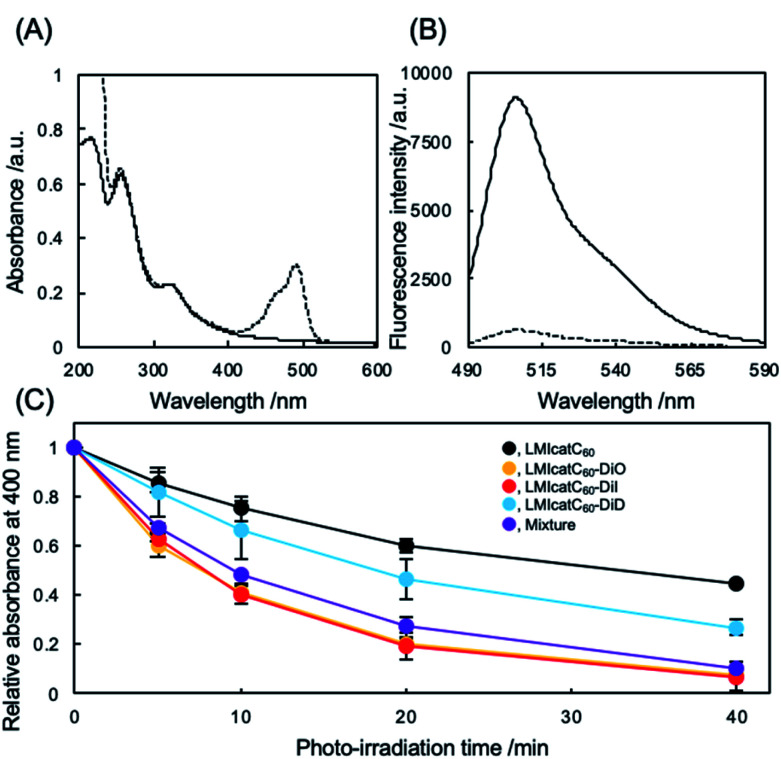
Photochemical properties of LMIcatC_60_–light-harvesting antenna molecules. (A) UV absorption spectra in an aqueous dispersion of LMIcatC_60_ (solid line) and LMIcatC_60_–DiO (dashed line) containing DMPC (1 mM), catC_60_ (0.05 mM), and DiO (0.025 mM). (B) Fluorescence spectra of an aqueous dispersion of LMIDiO (solid line) and LMIcatC_60_–DiO (dashed line) containing DMPC (1 mM), catC_60_ (0.05 mM), and DiO (0.025 mM). (C) Time course for the bleaching of 9,10-anthracenediyl-bis(methylene)-dimalonic acid (ABDA) *via* oxidation by singlet oxygen molecules. A DMSO solution of ABDA (25 μM) was added to the dispersion of liposome containing catC_60_ and the photo-antenna molecules (DMPC, 100 μM; catC_60_, 2.5 μM; photo-antenna molecules, 2.5 μM) followed by white light irradiation (>300 nm, 15 mW cm^−2^). The three independent experiments were conducted in duplicate.

**Table 1:** Basic physicochemical and biological properties of LMIcatC_60_–light-harvesting molecules

	(*D*_H_/nm)^a^	PDI^a^	ζ-Potential^b^/(mV)	Quenched dye/(%)	(MBC_90_/μg mL^−1^)^c^	(MIC/μg mL^−1^)^d^	Hemolysis^e^/(%)
LMIcatC_60_	55 ± 2	0.16	+45 ± 3	—	n.d.	n.d.	1.6 ± 0.1
LMIcatC_60_–DiO	55 ± 3	0.18	+34 ± 4	94	5.1<	29.3<	n.d.
LMIcatC_60_–DiI	60 ± 3	0.12	+42 ± 2	93^g^	2.4	1.25	1.2 ± 0.1
LMIcatC_60_–DiD	n.d.^f^	n.d.	n.d.^f^	87^g^	3.2<	1.25	2.5 ± 0.1
Mixture	n.d.^f^	n.d.	n.d.^f^	—	1.2	1.25	n.d.

a The hydrodynamic diameter was determined by conducting dynamic light scattering (DLS) measurements in Milli-Q water (25 °C; pH, 7.4). The PDI value was calculated using the cumulant method from an auto-correlation curve.

b The ζ potential was determined using capillary cells.

c MBC_90_ is defined as the concentration of the antimicrobial agent required to kill 90% of the bacterial colony within 1 h with irradiation.

d MIC is defined as the minimum inhibitory concentration of bacterial growth with irradiation using white light.

e Lysis of red blood cells was determined at 2.5 μM and carried out under irradiation.

f The absorption of DiD interfered with the DLS instruments.

g The quenching efficiencies of LMIcatC_60_–DiI and LMIcatC_60_–DiD were previously reported.^32^

To evaluate whether our systems were suitable photosensitizers, the quantity of ^1^O_2_ generated by our system under white light irradiation (>400 nm) was determined. Initially, we conducted a stability check against photo-irradiation by measuring the UV-vis spectra. As shown in Fig. S2,† catC_60_ showed excellent stability in all the systems against photo-irradiation under the current conditions. In contrast, photobleaching was observed in all the systems (Fig. S3†). After 40 min of irradiation, the absorbance at the maximum absorption wavelength from DiO of LMIcatC_60_–DiO (490 nm), DiI of LMIcatC_60_–DiI (552.5 nm), and DiD of LMIcatC_60_–DiD (648 nm) decreased to 53%, 90%, and 45%, respectively (Fig. S4†). Similar trends were observed in all dyes in the case with the mixture of these three systems. Furthermore, we examined the colloidal stability during photo-irradiation using DLS. The results revealed that the hydrodynamic diameter and PDI value did not significantly change *via* photo-irradiation. A time course of ^1^O_2_ generation was established by monitoring the conversion of the anthracene derivative 9,10-anthracenediyl-bis(methylene)dimalonic acid (ABDA) into endoperoxide (Scheme S2†).^35^ As ^1^O_2_ oxidized ABDA, the maximum absorbance peaks at 360, 380, and 400 nm that were characteristic of ABDA decreased as shown in Fig. S5.† The ratio of absorbance of ABDA at 400 nm, which was expressed as the amount of ABDA at each time point relative to the total concentration of ABDA, was quantified as a measure of the system's ability to generate ^1^O_2_ under white light irradiation in an O_2_-saturated aqueous solution (Fig. 2C). The LMIDiD, catC_60_-free control systems, did not generate ^1^O_2_ under irradiation with white light (Fig. S5A†). The ^1^O_2_ expression level of catC_60_ (Fig. 2C, black line) was accelerated by combining it with photo-harvesting antenna molecules, which indicated that the energy transfer process from the photo activated photo-antenna molecules to catC_60_ was efficient. When comparing the photodynamic activity of these LMIcatC_60_–dyad systems, the LMIcatC_60_–DiO and LMIcatC_60_–DiI systems generated the largest amount of ^1^O_2_*via* photo irradiation under the prevailing conditions (ABDA, 25 μM; lipids, 1 mM; catC_60_, 2.5 μM; light-harvesting molecules, 1.25 μM) (Fig. 2C, yellow and red lines, respectively). In contrast, the photodynamic activity of the LMIcatC_60_–DiD system was lower than that of the other dyad systems (Fig. 2C, blue line). These results suggested that enhancing the photodynamic activities of the LMIcatC_60_ systems was highly dependent on the activation frequencies of the light-harvesting antenna molecules attained *via* irradiation. These differences were observed in the quenching efficiencies (Table 1). To expand the absorption capacity for white light across a wide range of wavelengths and improve the availability of catC_60_, we examined a mixture of these three dyes with the same concentration of total catC_60_ as that used for the other dyad systems (lipids, 1 mM; catC_60_, 0.05 mM; DiO, 0.08 mM; DiI, 0.08 mM; DiD, 0.08 mM; Fig. 2C, purple line). We found that they all exhibited the same level function as the LMIcatC_60_–DiO and LMIcatC_60_–DiI systems.

The antibacterial activity of our systems was tested against *Escherichia coli* (*E. coli*) as a measure of their photodynamic character. Under the prevailing conditions, the nonionic LMI catC_60_–DiD system did not work well as an antibacterial agent even during irradiation (Fig. S6 and S7†). It was theorized that the electrically negative character of *E. coli* (approximately −15 mV) prevented maximum interaction between the dyad systems and the *E. coli* colony (Fig. S8†). To enhance the interaction of dyad systems with *E. coli*, cationic lipids (2) were prepared using the aforementioned method and tested; the solution properties of these lipids, including their hydrodynamic diameter and ζ potential, are summarized in Table 1 (1, 45 mM; 2, 5 mM). Unfortunately, both the hydrodynamic diameter and the ζ potential of samples containing DiD could not be measured accurately since its absorption interfered with the DLS instrument. We addressed the morphological observation of our systems using transmission electron microscopy; the analysis revealed a spherical morphology with a lipid-bilayer. The size of our dyad systems agreed with the results of the DLS measurements (Fig. S9†). In addition, it was revealed that the diameter of LMIcatC_60_–DiD is similar to that of the other systems. Thus, the antimicrobial activity was established based on the photodynamic character of our LMIcatC_60_–light-harvesting antenna molecule using the cationic lipids. Initially, the minimum bactericidal concentration (MBC_90_), which is defined as the lowest concentration of the antibacterial agent needed to destroy 90% of a particular bacterial colony in broth media, was determined.^36^ Briefly, after co-incubation with our dyad systems for 30 min, the *E. coli* cells were irradiated with white light for 30 min. A 24 h incubation period then followed, after which the viability of the *E. coli* was estimated by measuring the optical density of the colony at 600 nm.

As shown in Fig. 3A, no apparent cytotoxicity against *E. coli* was established for the “dark” (no irradiation) conditions. In contrast, antimicrobial activities were obtained under irradiation except for the LMIcatC_60_ systems in which the concentration of catC_60_ was between 0 and 2.5 μM (Fig. 3B). The MBC_90_ values for the LMIcatC_60_–DiO, LMIcatC_60_–DiI, LMIC_60_–DiD, and dyad mixture were 5.1, 2.4, 3.2, and 1.2 μM, respectively (Table 1). As expected, the LMIcatC_60_–light-harvesting molecules enhanced the antimicrobial activity of catC_60_. Moreover, the system containing a mixture of LMIcatC_60_–DiO, LMIcatC_60_–DiI, and LMIcatC_60_–DiD showed the highest antimicrobial activity, whereas the lowest antimicrobial activity was associated with the LMIcatC_60_–DiO dyad despite its excellent ^1^O_2_ production capacity. The reason for this is discussed in the next paragraph. To address their availability as photoinduced antimicrobial agents, the minimal inhibitory concentration was investigated using an agar-based colony-forming assay (Fig. S10–S14†). Here similar trends in the dyads' antimicrobial profile were found (Fig. 3C and D),^36^ namely, colony formation was drastically suppressed even at low concentrations (catC_60_ <1.25 μM) upon exposure to LMIcatC_60_–DiI, LMIC_60_–DiD, and the dyad mixture with irradiation (400–800 nm). The MIC value of our systems is summarized in Table 1; here, it was noted that the antimicrobial activity of the DiO systems was quite low when compared with the other catC_60_–photo-harvesting antenna molecule assembled systems. We hypothesized that these differences in performance were caused by variations in the extent of the interaction and the uptake of the appropriate concentrations.

**Fig. 3:**
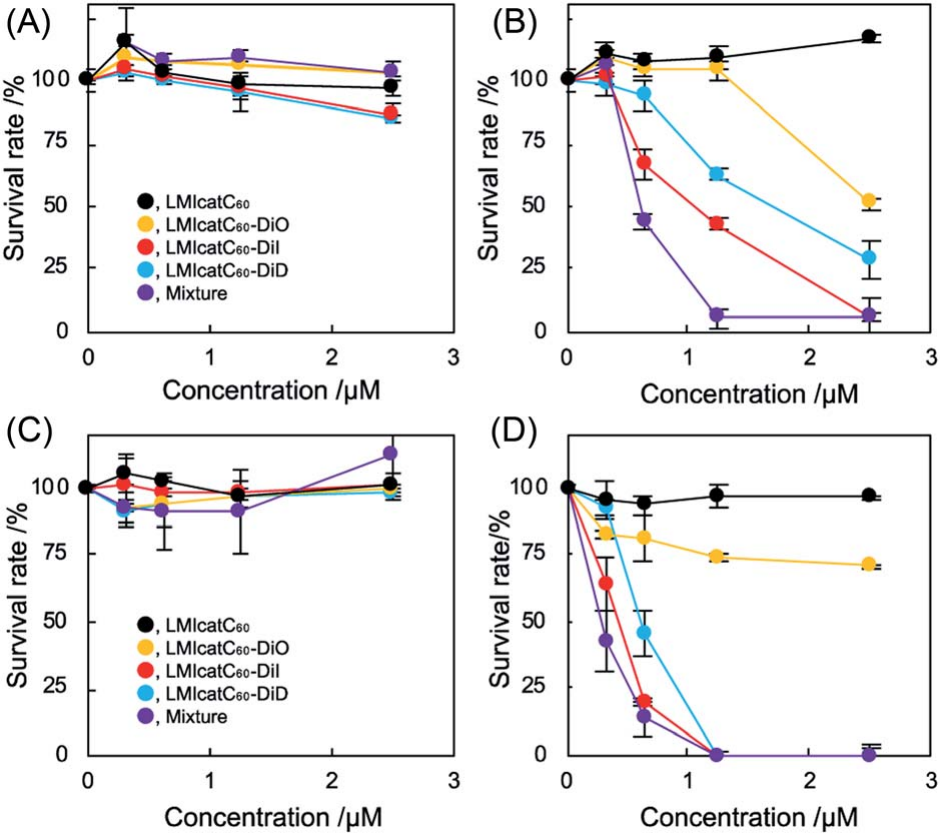
The antimicrobial activity of LMIcatC_60_–light-harvesting molecules under photo irradiation. (A and B) Minimal bactericidal concentration assay of the fullerene/photo-antenna molecule–liposome hybrid system without irradiation (A) and with irradiation (B) against *E. coli*. The *E. coli* (2 × 10^7^ cfu) cells were co-incubated with the fullerene/photo-antenna molecule–liposome hybrid for 30 min, after which they were irradiated (300–740 nm) for 30 min. After a 24 h incubation period in the culture media, the survival rate was quantified by measuring the optical density at 600 nm using a microplate reader. Three independent experiments were conducted in duplicate. (C and D) Minimal inhibitory concentration assay of the fullerene/photo-antenna molecule–liposome hybrid system without irradiation (C) and with irradiation (D) against *E. coli*. The *E. coli* (4 × 10^5^ cfu) cells were co-incubated with the fullerene/photo-antenna molecule–liposome hybrid for 30 min, after which they were subjected to irradiation (300–740 nm) for 30 min. After incubation for 24 h on MHB agar gel, the survival rate was quantified. The three independent experiments were conducted in duplicate.

To verify our hypothesis, interaction of the LMI system with *E. coli* was studied using confocal laser scanning microscopy (Fig. 4). After co-incubation with LMIDiO, LMIDiI, LMIDiD (lipids, 1 mM; light-harvesting antenna molecules, 25 μM), and the mixture (lipids, 1 mM; DiO, 8.3 μM; DiI, 8.3 μM; DiD, 8.3 μM), liposomes containing the dyes were detected in the *E. coli* (Fig. 4B, D, F, and H–J). Fluorescence signals for DiO were quite faint compared with the signals from the other systems; however, these signals were much brighter when the mixture was used regardless of the extent of dilution. Thus, the strong interactions of our systems with *E. coli* enhanced the photoinduced damage caused by the ^1^O_2_ molecules that had been generated *via* photodynamic activity. These differences in the extent of the interaction between the system and the bacteria may be caused by the electrostatic character of the respective systems.

**Fig. 4:**
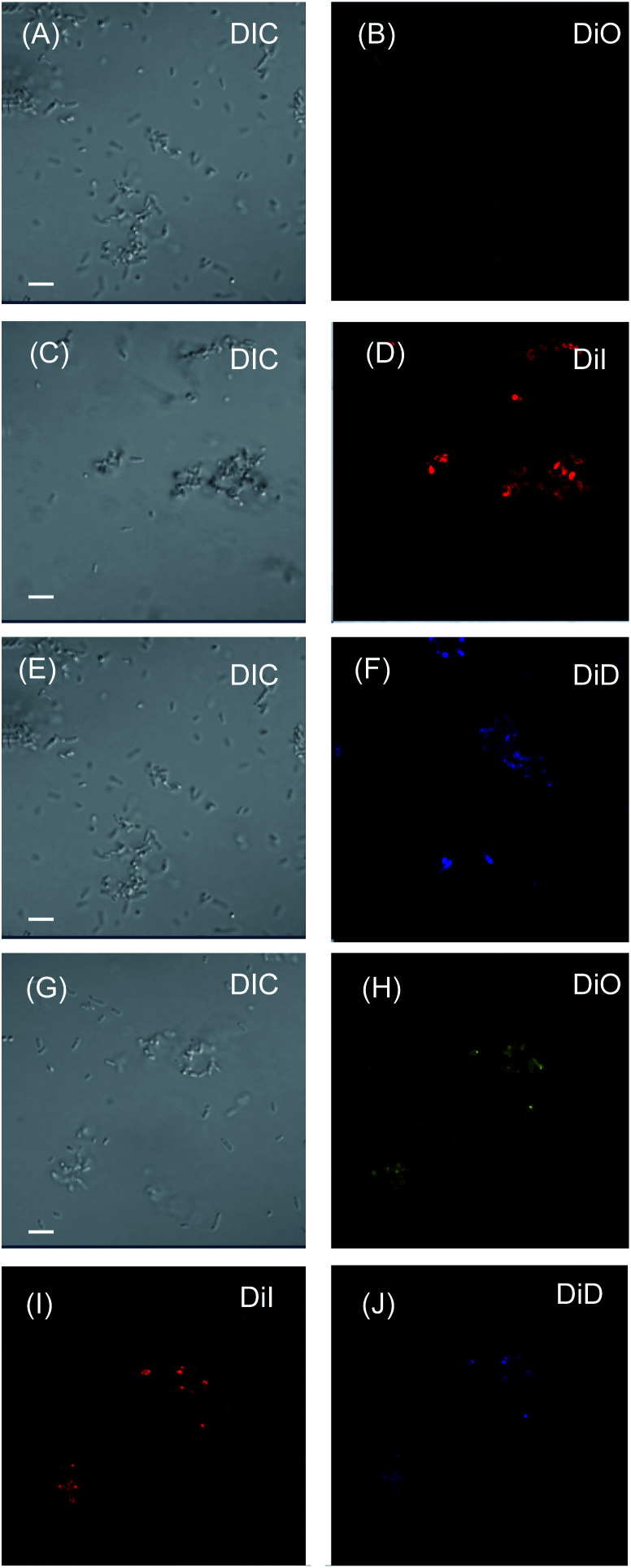
Interaction of LMIDiO (A and B), LMIDiI (C and D), LMIDiD (E and F), and the dyad mixture systems (G–J) with *E. coli.* Here, the *E. coli* cells (4 × 10^5^ cfu) were co-incubated with the dye-liposome hybrid for 30 min (dye, 15 μM). The treated *E. coli* colonies were observed using confocal laser scanning microscopy. The scale bar represents 20 μm.

To determine the hemolytic effect of each system, the lytic activity against red blood cells (RBCs)^36^ was quantified after a 1 h period of exposure of a verified concentration of the dyad systems to RBCs by measuring the absorbance of the leaked myoglobin. No apparent lytic activity was observed even at the highest concentration of the dyad after irradiation with white light (Table 1 and Fig. S15†), suggesting that our system was a potentially safe alternative for eliminating bacterial content under white light irradiation.

In conclusion, we present a facile method for the elimination of bacteria based on the photodynamic activity of cationic fullerene–photo-harvesting antenna molecules integrated into a liposome membrane. We were able to expand the absorption capacity of these fullerene derivatives mainly *via* the development of more efficient energy transfer processes from the photo activated antenna molecules to the fullerene derivatives. As noted in this study, our strategy has great potential for use in the photo dynamically influenced elimination of bacterial content.

## Conflicts of interest

There are no conflicts to declare.

## Supplementary Material

NA-002-D0NA00132E-s001
