# Supplementary material for: Bacterial elimination via the photodynamic activity of a fullerene/light-harvesting antenna molecule assembled system integrated into liposome membranes
Source: Nanoscale Adv. 2020 Aug 25;2(10):4395–9. doi: 10.1039/d0na00132e (PMC9418483; doi:10.1039/d0na00132e)
Supplement: NA-002-D0NA00132E-s001 [file NA-002-D0NA00132E-s001.pdf]

Electronic Supplementary Material (ESI) for Nanoscale Advances.
This journal is © The Royal Society of Chemistry 2020

Supplemental Information for

Bacterial elimination system based on photodynamic activity using fullerene/light-harvesting antenna molecule assembled system integrated in liposome

Riku Kawasaki*, Daiki Antoku, Reo Ohdake, Kouta Sugikawa, Atsushi Ikeda*

Department of applied chemistry, Graduated school of engineering, Hiroshima university, 1-4-1 Kagamiyama, Higashi Hiroshima, 739-8527, Japan

Materials and Methods

Materials

C_{60} were bought from MER Co. (Tucson, AZ). γ-CDx and ABDA was purchased from Fuji Film (Tokyo, Japan) and Sigma Aldrich (Milwaukee, WI, USA). 1,1'-dioctadecyl-3,3,3',3'-tetramethylindocarbo-cyanine perchlorate (DiI), 1,1'-dioctadecyl-3,3,3',3'-tetramethylindodicarbocyanine (DiD), and 3,3'-dioctadecyloxacarbocyanine perchlorate (DiO) were purchased from Molecular Probes, Inc. (Eugene, OR, USA). *E. coli* and red blood cells were obtained from ATCC (VA, USA) and Funakoshi (Tokyo, Japan). The *E. coli* were maintained in broth media at 37 ^{o}C in shaking incubator. MIB and MIC were purchased from Sigma Aldrich.

Preparation of LIMcatC_{60}-light harvesting molecule (DiI, DiD)

LIMcatC_{60}-DiI and DiD were prepared as previously reported method. Lipid **1** (50 mM) or mixture of **1** and **2** (**1**, 45 mM; **2**, 5 mM) were dissolved in chloroform. DiI and DiD were dissolved in methanol. These two solutions were mixed and the solvents were vapored via flowing a gentle stream of nitrogen. The resulting thin films were hydrated above the phase transition temperature (Lipids, 2 mM; DiI or DiD, 0.05 mM). To obtain ulilamellar vesicles, freeze-thaw were carried out at least five times (-195 and 50 ^{o}C). Afterward, the resulting dispersion were treated to uniform in size using extruder with 0.05 μm pore filters with heating above transition temperature. The liposome dispersion obtained were mixed with catC_{60}/γ-CDx complex (Lipids, 1 mM; DiI or DiD, 0.025 mM; catC_{60}, 0.05 mM) prepared via high-speed vibration grinding method using ball mill mixer. To obtain LIMcatC_{60}-DiI or LIMcatC_{60}-DiD via exchanging reaction, the resulting mixtures were heated above transition temperature (80 ^{o}C) with stirring for 2 h. The complexation of catC_{60} were confirmed by measuring UV-Vis spectra (UV-3600 spectrophotometer; Shimadzu Corporation, Tokyo, Japan) and fluorescence spectra (excitation; DiI, 549 nm; DiD, 644 nm) (F-4500 fluorescence spectrophotometer; Hitachi Ltd, Tokyo, Japan).

Preparation of LIMcatC$_{60}$-light harvesting molecule (DiO)

After preparation of LIMcatC$_{60}$ via exchanging reaction via exchanging reaction, DiO dissolved in DMSO were injected toward the dispersion (Lipids, 1 mM; DiO, 0.025 mM; catC$_{60}$, 0.05 mM). Formulation of LIMcatC$_{60}$-DiO were confirmed by measuring UV-Vis spectra and fluorescence spectra (DiO, 484 nm).

Basic characterization of LMIcatC$_{60}$-light-harvesting antenna molecules

The hydrodynamic diameters and the zeta-potentials of LMIcatC$_{60}$-light harvesting molecules were measured by using dynamic light scattering and capillary cells (Zetasizer Nano ZS, Malvern Instruments, UK). The samples were placed on the grid and dried up overnight. Afterward, the samples were stained with 1.5% ammonium molybdate for 10 s. Morphological observation was carried out using transmission electron microscope (JEM-1400, JEOL Ltd., Tokyo, Japan).

Stability of LMIcatC$_{60}$-light harvesting antenna molecule systems against photo-irradiation.

LMIcatC$_{60}$-light harvesting antenna molecule systems (Lipids, 1 mM; catC$_{60}$, 2.5 μM; light-harvesting molecules, 1.25 μM) Oxygen gas was bubbled through all the sample dispersions for 30 min before irradiation. White light was irradiated against the resulting dispersion using a xenon lamp (SX-UID500X, 500W; Ushio Inc., Tokyo, Japan). The power of the light was fixed at 16 mW cm^{-2}. At each time point (0, 5, 10, 20, and 40 min), the absorbance spectra of the dispersion were measured from 300 to 600 nm. In addition, DLS measurements were carried out.

ABDA photo-bleaching measurement

Relative amount of the $^1O_2$ generated via white light irradiation were quantified with ABDA bleaching method. ABDA solution dissolved in DMSO were applied to dispersion of LMIcatC$_{60}$, LMIcatC$_{60}$-DiO, LMIcatC$_{60}$-DiI, LMIcatC$_{60}$-DiD, or mixture of LMIcatC$_{60}$-DiO, LMIcatC$_{60}$-DiI, and LMIcatC$_{60}$-DiD (ABDA, 25 μM; Lipids, 1 mM;

catC$_{60}$, 2.5 μM; light-harvesting molecules, 1.25 μM). Oxygen gas was bubbled through all the sample dispersions for 30 min before irradiation. White light was irradiated against the resulting dispersion using a xenon lamp (SX-UID500X, 500W; Ushio Inc., Tokyo, Japan). The power of the light was fixed at 16 mW cm^{-2}. At each time point (0, 5, 10, 20, and 40 min), the absorbance spectra of the dispersion were measured from 300 to 600 nm.

MBC assay

The minimum bactericidal concentration (MBC) was determined as the lowest concentration of catC$_{60}$ to eliminate a particular bacterium, *E. coli* here. MBC$_{90}$ of catC$_{60}$ against *E. coli* were determined as the concentration in killing 90% of bacterium in PBS. *E. coli* were maintained in Mueller Hinton Broth (MHB) overnight to obtain exponential phase (OD$_{600}$, 0.5-0.7). *E. coli* were re-suspended in PBS at 2×10^7 cfu/mL (OD$_{600}$, 0.05). Dispersion of LMIcatC$_{60}$, LMIcatC$_{60}$-DiO, LMIcatC$_{60}$-DiI, LMIcatC$_{60}$-DiD, or mixture of LMIcatC$_{60}$-DiO, LMIcatC$_{60}$-DiI, and LMIcatC$_{60}$-DiD (Lipids, 1 mM; catC$_{60}$, 2.5 μM; light-harvesting molecules, 1.25 μM) were added to the resulting *E. coli* suspension and incubate for 30 min. Then, white light irradiation was carried out toward the resulting mixture for 30 min. The suspension diluted with PBS (1000-fold) were streaked on Muller Hinton agar plates, and incubated at 37 ^{o}C overnight. The colony formulated were counted as colony forming unit.

MIC assay

The minimum inhibitory concentration (MIC) was determined by standard microbroth dilution assay. *E. coli* were maintained in Mueller Hinton Broth (MHB) overnight to obtain exponential phase (OD$_{600}$, 0.5-0.7). *E. coli* were re-suspended in MHB at 4×10^5 cfu/mL. Dispersion of LMIcatC$_{60}$, LMIcatC$_{60}$-DiO, LMIcatC$_{60}$-DiI, LMIcatC$_{60}$-DiD, or mixture of LMIcatC$_{60}$-DiO, LMIcatC$_{60}$-DiI, and LMIcatC$_{60}$-DiD (Lipids, 1 mM; catC$_{60}$, 2.5 μM; light-harvesting molecules, 1.25 μM) were added to the resulting *E. coli* suspension and incubate for 30 min. Then, white light irradiation was carried out toward the resulting mixture for 30 min. The resulting suspension were incubated at 37 ^{o}C

overnight and their optical density was measured by microplate reader.

Hemolysis assay

Goat red blood cells were re-suspended in PBS (3×10^8 cells/mL) and co-incubated with LMIcatC$_{60}$, LMIcatC$_{60}$-DiO, LMIcatC$_{60}$-DiI, LMIcatC$_{60}$-DiD, or mixture of LMIcatC$_{60}$-DiO, LMIcatC$_{60}$-DiI, and LMIcatC$_{60}$-DiD (Lipids, 1 mM; catC$_{60}$, 2.5 μM; light-harvesting molecules, 1.25 μM) for 30 mins. Afterward, white light irradiation was carried out against resulting mixture. Triton-X100 (1%) was used as the positive lysis control and PBS was used as negative control. After incubation, the suspensions were centrifuged at 1000g for 5 min and the absorbance at 410 nm from hemoglobin released from RBC were measured by microplate reader.

Supplemental Figures

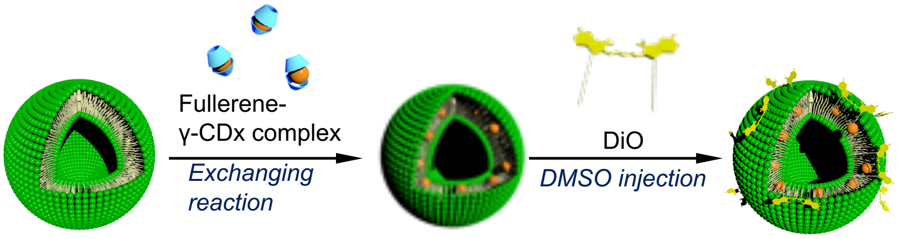

Scheme S1. Preparation of catC_{60}-DiO assembled in liposome via DMSO injection method.

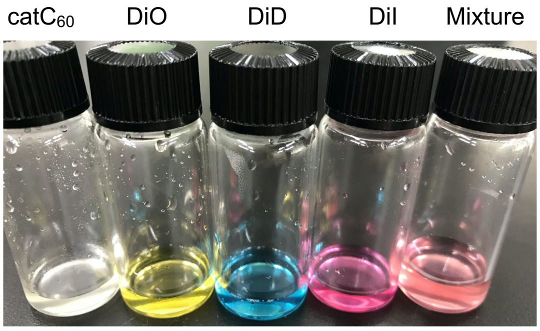

Fig. S1. Representative photograph of fullerene/photo-antenna molecule-liposome hybrid

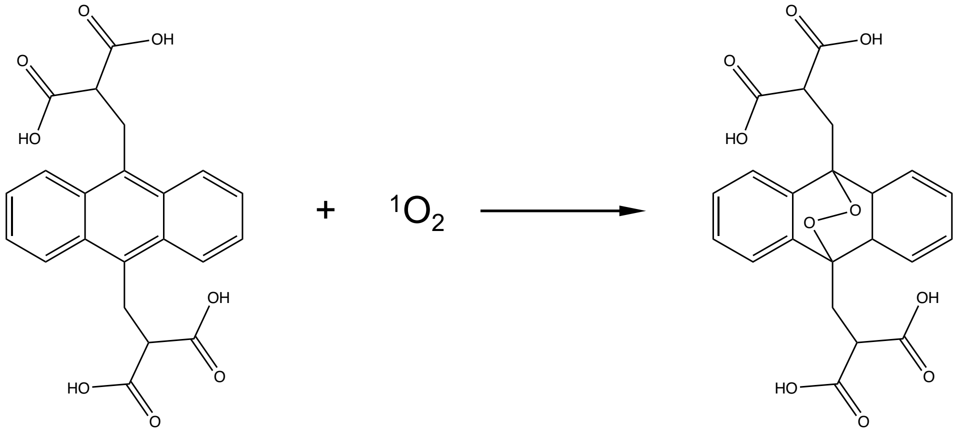

Scheme S2. Conversion of ABDA to endoperoxide via oxidation by $^{1}O_2$.

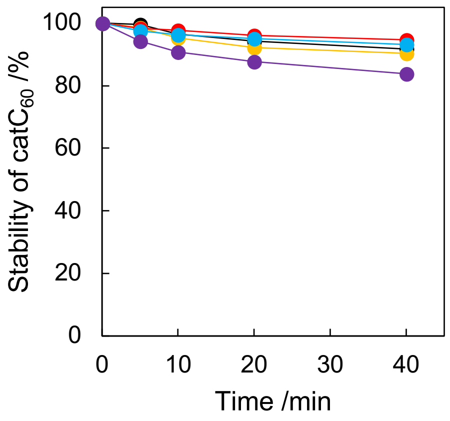

Fig. S2. Stability of $catC_{60}$ in $LMIcatC_{60}$-light harvesting molecule systems against photo-irradiation. Dispersion of liposome containing $catC_{60}$ and photo antenna molecules (DMPC, 100 μM; $catC_{60}$, 2.5 μM; photo antenna molecules, 2.5 μM). were irradiated with white light (>300 nm, 15 mW cm^{-2}).

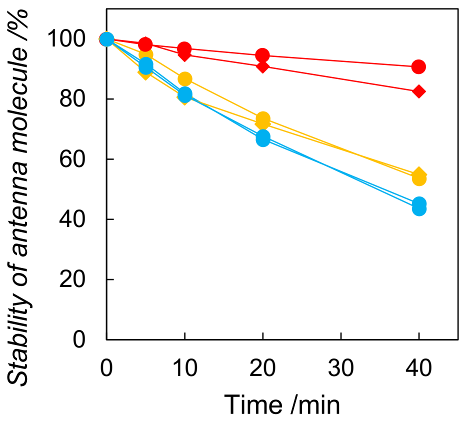

Fig. S3. Stability of light-harvesting antenna molecules in $LMIcatC_{60}$-light harvesting molecule systems against photo-irradiation. Dispersion of liposome containing $catC_{60}$ and photo antenna molecules (DMPC, 100 μM; $catC_{60}$, 2.5 μM; photo antenna molecules, 2.5 μM). were irradiated with white light (>300 nm, 15 mW cm^{-2}). DiO (yellow; $LMIcatC_{60}$-DiO, circle; Mixture, diamond), DiI (red; $LMIcatC_{60}$-DiI, circle; Mixture, diamond), and DiD (blue; $LMIcatC_{60}$-DiD, circle; Mixture, diamond).

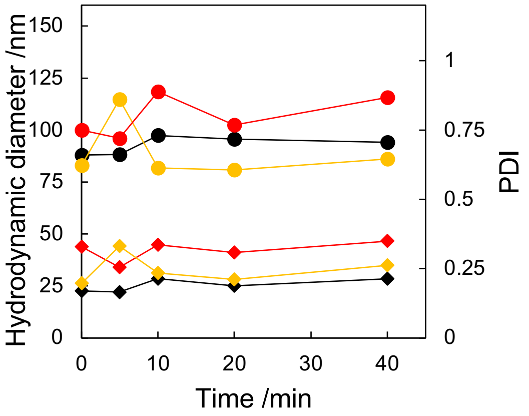

Fig. S4. Size changes of LMIcatC_{60}-light harvesting antenna molecule systems during light irradiation. LMIcatC_{60}(black), LMIcatC_{60}-DiO (yellow), and LMIcatC_{60}-DiI (red). Circle and diamond represents hydrodynamic diameter and PDI, respectively.

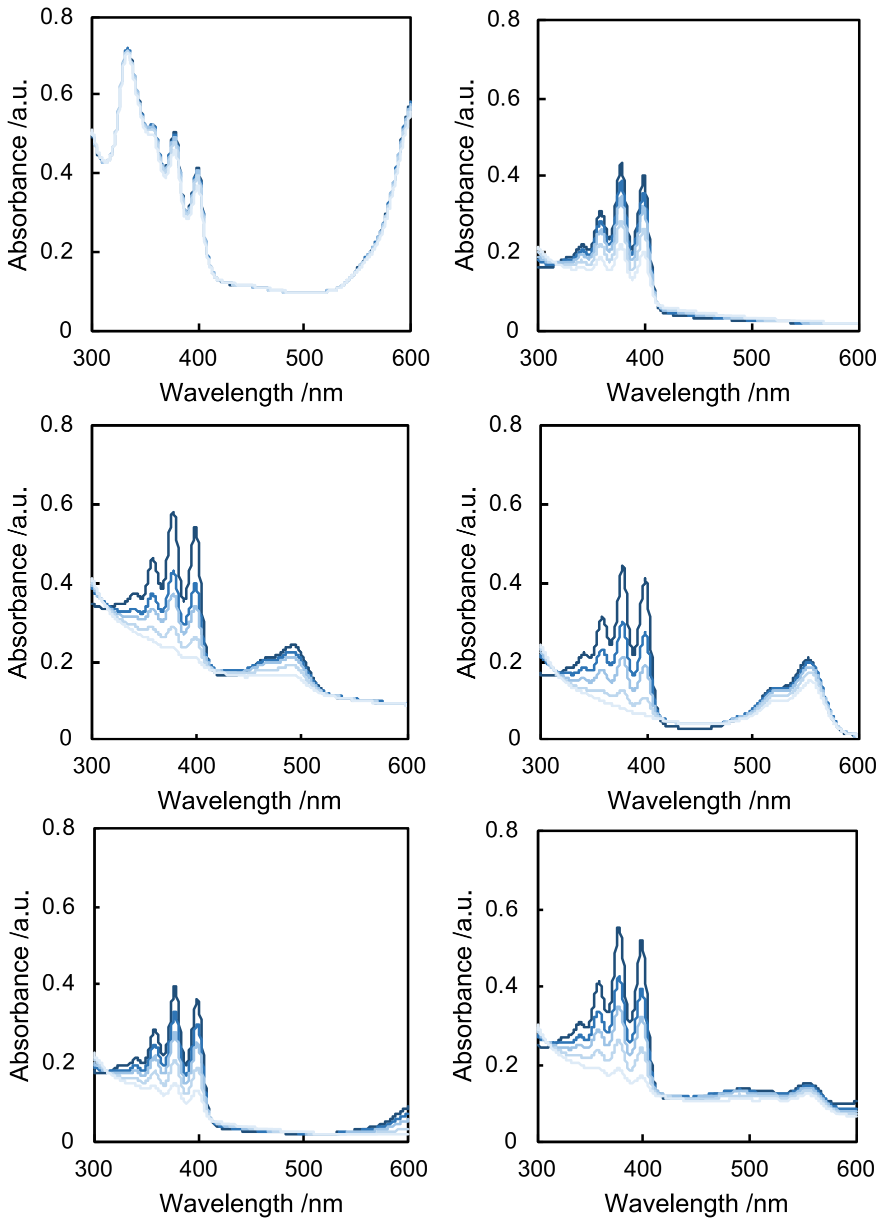

Fig. S5. Representative UV absorption spectrum in bleaching of ABDA via oxidation with $^1O_2$ generated by DiD/liposome (A), $catC_{60}$/liposome (B), $catC_{60}$-DiO/liposome (C), $catC_{60}$-DiI/liposome (D), $catC_{60}$-DiD/liposome (E), mixture of $catC_{60}$-DiO/liposome, $catC_{60}$-DiI/liposome, and $catC_{60}$-DiD/liposome (F) with white light irradiation (> 300 nm, 15 mW cm^{-2}). The measurements were carried out at each time point (0, 5, 10, 20 and 40 min). (DMPC, 100 μM; $catC_{60}$, 2.5 μM; photo antenna molecules, 2.5 μM).

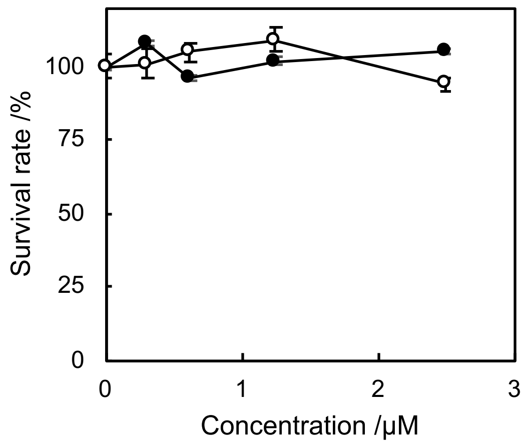

Fig. S6. Minimal bactericidal concentration assay of $catC_{60}$ DiD Liposome without irradiating light (closed circle) and with irradiating light (open circle) .

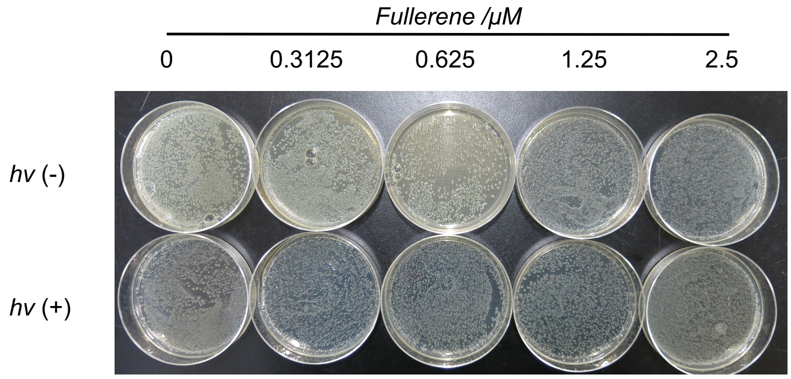

Fig. S7. Representative photograph of plate containing *E. coli* on MHB treated with $catC_{60}$-DiD/liposome with or without irradiating light

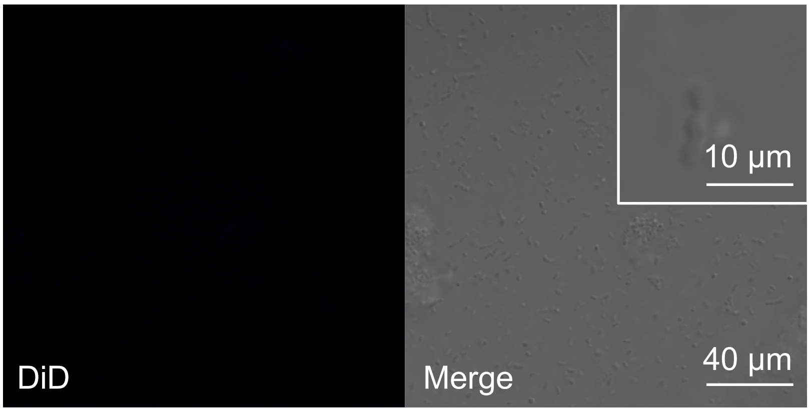

Fig. S8. Representative fluorescent images of *E. coli* treated with DiD containing liposome.

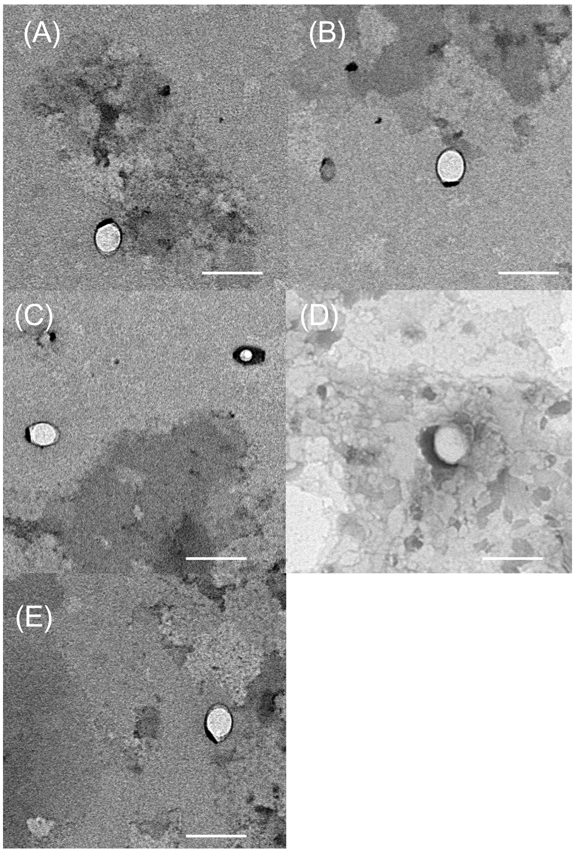

Fig. S9. Representative morphologies of LMIcatC$_{60}$-light harvesting antenna molecule system. LMIcatC$_{60}$ (A), LMIcatC$_{60}$-DiO (B), LMIcatC$_{60}$-DiI (C), LMIcatC$_{60}$-DiD (D), and Mixture (E). The samples were stained with 1.5% ammonium molybdate. Error bar represents 200 nm.

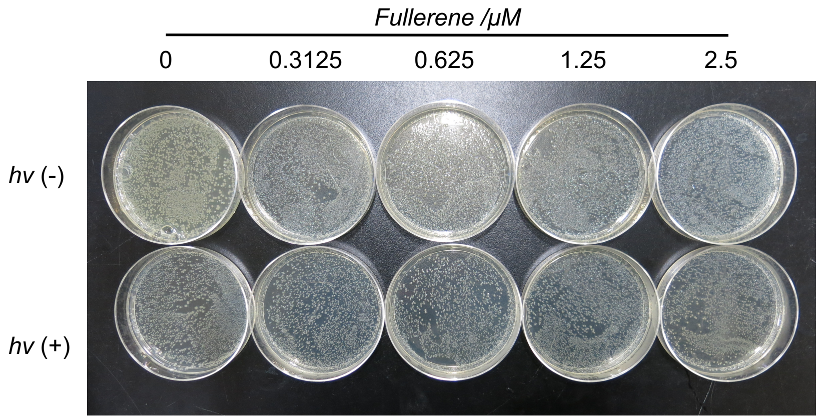

Fig. S10. Representative photograph of plate containing *E. coli* on MHB treated with fullerene/liposome hybrid with or without irradiating light

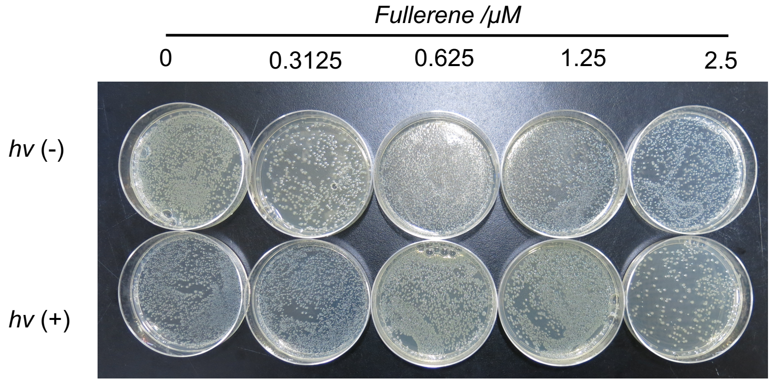

Fig. S11. Representative photograph of plate containing *E. coli* on MHB treated with fullerene-DiO/liposome hybrid with or without irradiating light

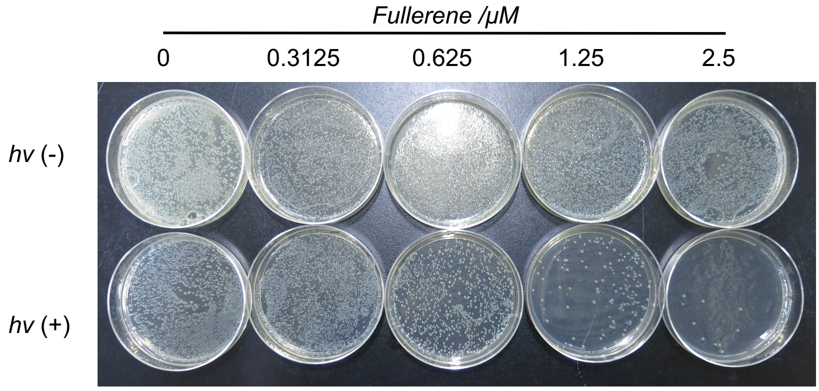

Fig. S12. Representative photograph of plate containing *E. coli* on MHB treated with fullerene-DiI/liposome hybrid with or without irradiating light

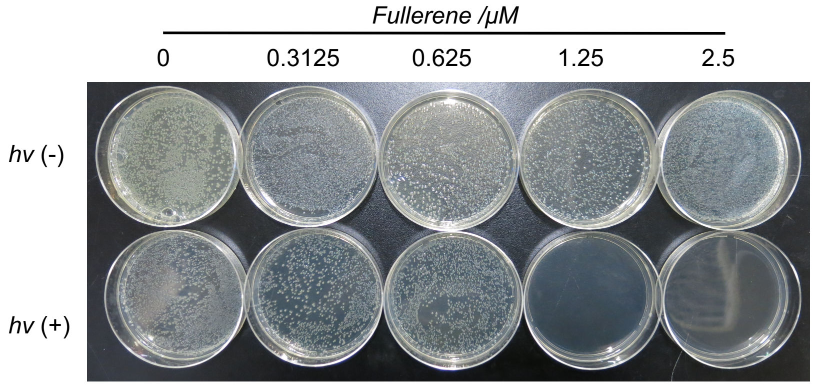

Fig. S13. Representative photograph of plate containing *E. coli* on MHB treated with fullerene-DiD/liposome hybrid with or without irradiating light

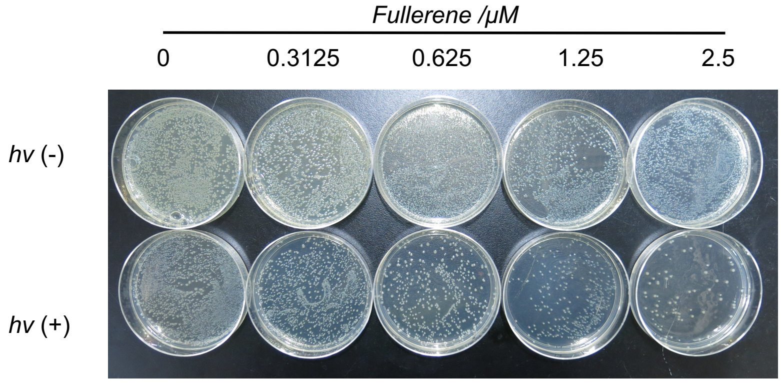

Fig. S14. Representative photograph of plate containing *E. coli* on MHB treated with fullerene-mixture of DiO, DiI, and DiD/liposome hybrid with or without irradiating light

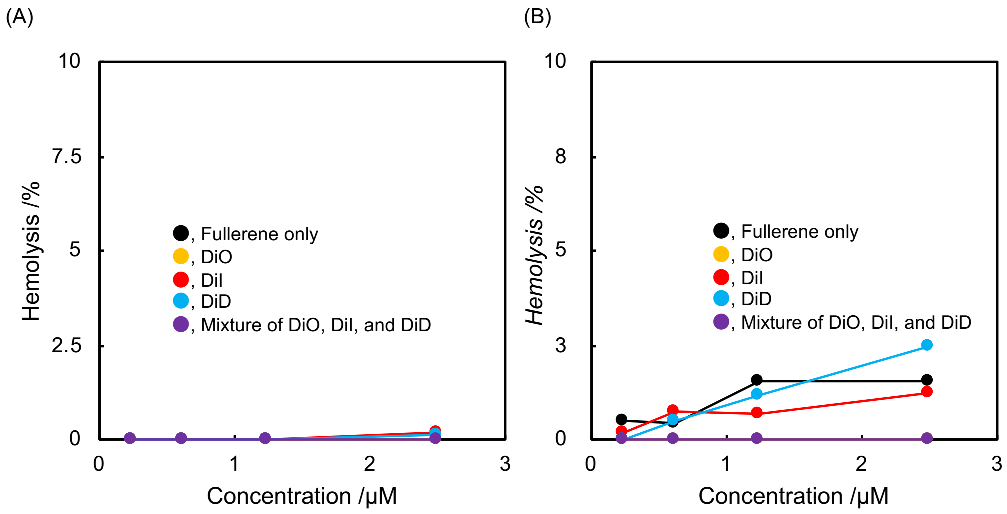

Fig. S15. Hemolysis assay without irradiation white light (A) and with irradiation white light (B). Red blood cells were co-incubated with catC$_{60}$-photo antenna molecule/liposome for 1 h with irradiating light. 10 % Triton-X for 3 h incubation was used as positive control. After centrifugation, the absorbance at 550 nm in the supernatant were measured by microplate reader. Three independent experiments were carried out duplicate.
